# The phenotypic predisposition of the parent in F1 hybrid is correlated with transcriptome preference of the positive general combining ability parent

**DOI:** 10.1186/1471-2164-15-297

**Published:** 2014-04-22

**Authors:** Gaoyuan Song, Zhibin Guo, Zhenwei Liu, Xuefeng Qu, Daiming Jiang, Wei Wang, Yingguo Zhu, Daichang Yang

**Affiliations:** 1 State Key Laboratory of Hybrid Rice and College of Life Sciences, Wuhan University, Luojia Hill, Wuhan 430072, Hubei Province, China

**Keywords:** General combining ability (GCA), Transcriptome profiling, Hybrid rice, Transcriptome and phenotype bias, Diallelic crosses

## Abstract

**Background:**

Sprague and Tatum (1942) introduced the concepts of general combining ability (GCA) and specific combining ability (SCA) to evaluate the breeding parents and F1 hybrid performance, respectively. Since then, the GCA was widely used in cross breeding for elite parent selection. However, the molecular basis of GCA remains to unknown.

**Results:**

We studied the transcriptomes of three varieties and three F1 hybrids using RNA-Sequencing. Transcriptome sequence analysis revealed that the transcriptome profiles of the F1s were similar to the positive GCA-effect parent. Moreover, the expression levels of most differentially expressed genes (DEGs) were equal to the parent with a positive GCA effect. Analysis of the gene expression patterns of gibberellic acid (GA) and flowering time pathways that determine plant height and flowering time in rice validated the preferential transcriptome expression of the parents with positive GCA effect. Furthermore, H3K36me3 modification bias in the *Pseudo-Response Regulators* (PRR) gene family was observed in the positive GCA effect parents and demonstrated that the phenotype and transcriptome bias in the positive GCA effect parents have been epigenetically regulated by either global modification or specific signaling pathways in rice.

**Conclusions:**

The results revealed that the transcriptome profiles and DEGs in the F1s were highly related to phenotype bias to the positive GCA-effect parent. The transcriptome bias toward high GCA parents in F1 hybrids attributed to H3K36me3 modification both on global modification level and specific signaling pathways. Our results indicated the transcriptome profile and epigenetic modification level bias to high GCA parents could be the molecular basis of GCA.

## Background

Selecting elite parents is of paramount importance in cross- and hybrid-breeding programs. The selection of parents from a phalanx of inbred lines, however, is extremely laborious and time-consuming and can be random. Adding to this complexity, parents with excellent agronomic traits do not always pass those traits on to their progeny. To evaluate breeding parents, Sprague and Tatum (1942) introduced the concepts of general combining ability (GCA) and specific combining ability (SCA), which allow the study and comparison of the performances of inbred lines in hybrid combination. GCA is used to designate the average performance of an inbred line in hybrid combination, and SCA is used to designate those cases in which certain combinations do relatively better or worse than would be expected on the basis of the average performance of the lines involved [1]. GCA and SCA effects have been successfully used as criteria to evaluate elite parents in conventional crossbreeding and the performance of hybrid combinations.

Since the introduction of the GCA concept in 1942, very limited genetic studies on GCA have been conducted even though GCA is widely used in breeding programs for evaluation of the parents in early generations [2-5]. A few quantitative genetic loci with GCA have been identified recently. Qu *et al.* analyzed the QTLs of 10 agronomic traits for GCA using recombinant inbred line (RIL) populations with three testers in three testcross populations and a backcross recombinant inbred line (BCRIL) population of rice [6]. They detected a large number of additive effects of QTL^GCA^ loci. Qi *et al.* found that several genetic loci responding for GCA and SCA for five yield-related traits using a set of testcrosses with introgression lines (ILs) of maize under different environmental conditions. Total of 56 significant QTL^GCA^ loci have been mapped [7]. These studies have revealed that GCA effects, like the traits, are genetically controlled. At the molecular level, however, how the phenotypes associated with GCA effects are passed on to the F1 hybrids remains unknown.

High-throughput genome-wide analysis approaches such as microarray analysis and next generation sequencing have been used in the study of phenotypes such as hybrid vigor [8-10]. Genome-wide gene expression profiles related to heterosis have been studied [11-14]. Stupar et al. studied the gene expression profiles between maize F1 hybrids and their parents, and approximately 75% of the differentially expressed genes showed additive expression patterns between F1 hybrids and parents [13]. They studied the genetic diversity and transcriptional variation with different maize hybrids and found that the genetic diversity was correlated with transcriptional variation, and little (less than 1%) of the gene expression in F1 hybrids was outside the parental range [13]. Wei *et al.* studied the gene expression profile between the super hybrid rice LYP9 and its parents and suggested that the differentially expressed genes might correlate with heterosis. Moreover, Riedelsheimer *et al*. have analyzed metabolic pathways in maize hybrids and were able to predict GCA scores using genome-wide association analysis [15]. They have used 285 crosses derived from the diverse inbred lines of maize with two testers and predicted their combining ability for seven biomass- and bioenergy related traits using 56,110 single nucleotide polymorphisms (SNPs) and 130 metabolites. The prediction accuracies were from 0.72 to 0.81 for SNPs and 0.60 to 0.80 for metabolites. A genome-wide analysis of the association between GCA and gene expression profiles has not been investigated. The contribution of the elite parents with GCA effects to their offspring also remains unexplored.

In the present study, we constructed a diallele crossing population with five rice parents and 10 F1 hybrids. We evaluated the GCA effects of three agronomic traits related to grain yield, heading date, plant height and grain number from five elite rice varieties. The results showed that 93–11 and Teqing (TQ) have positive GCA effects, and Guangluai 4# (GL), Aijiaonante (AJ) and Zhenshan 97 (ZS) showed negative GCA effects. The analysis of the transcriptome profiles of the leaves from three F1 hybrids, GL × 93-11, GL × TQ and 93-11 × TQ, and their parents revealed that transcriptome profiles were correlated to the positive-GCA-effects parent, showing obvious parental bias. Further analysis found that the expression levels of most of the DEGs were obviously biased towards to the positive GCA effect parent. Analysis of the gene expression patterns of gibberellic acid (GA) and flowering-time signaling pathways for plant height and flowering time validated the transcriptome bias to the positive GCA effect parents. Our results also indicated that the H3K36me3 modification showed bias to the positive GCA effect parent and demonstrated that phenotypes of the positive GCA effect parents were attributed to transcriptome bias. These results will be helpful in understanding the parental gene contribution to F1 hybrids and the molecular basis of GCA.

## Results

### Evaluation of general combining ability of elite rice varieties

**Figure 1:**
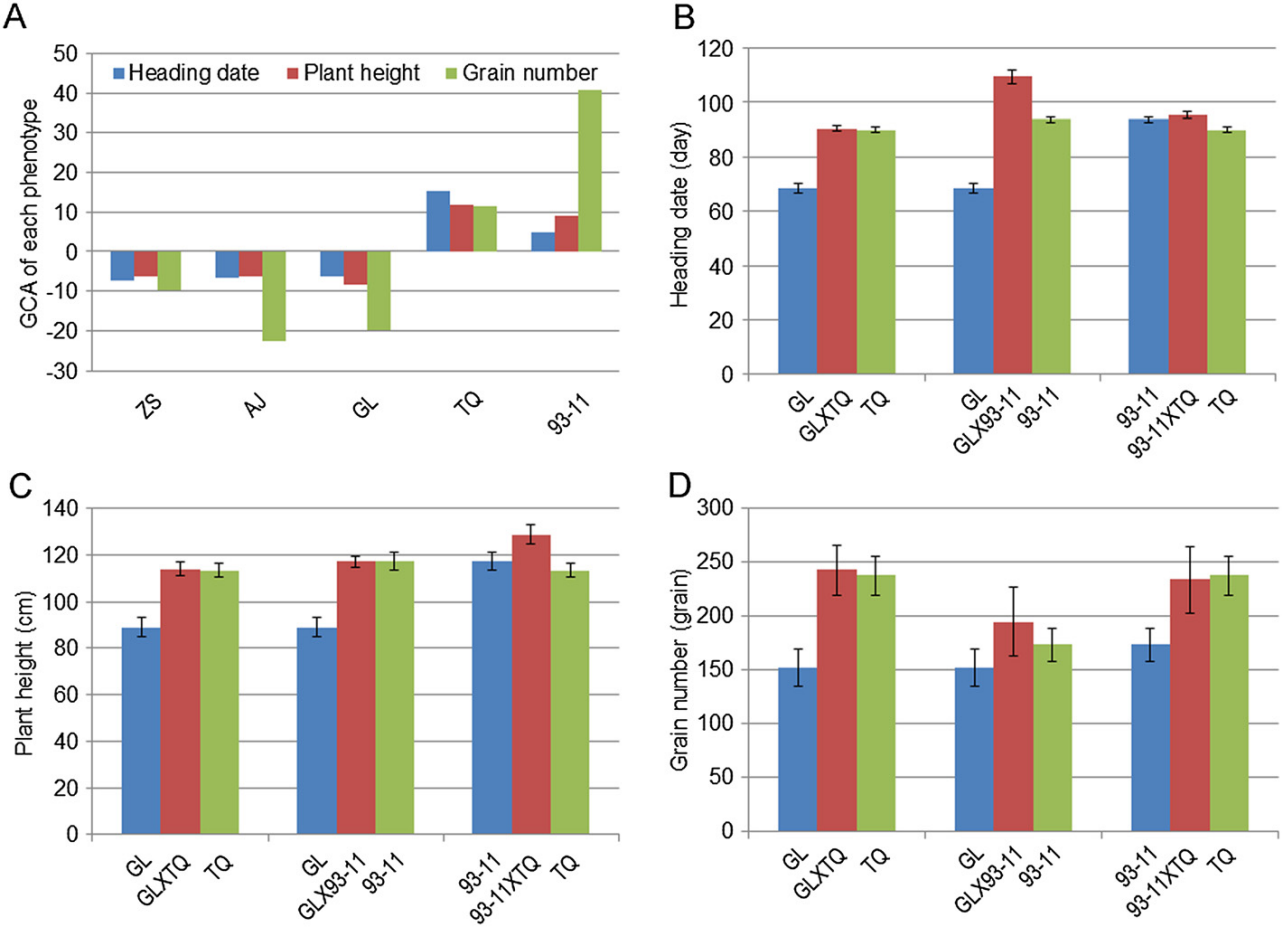
**The GCA scores and plant phenotypes of plant height, flowering time and plant spikelet per panicle from three F1 hybrids and their parents. (A)** GCA effect; **(B)** spikelet number per panicle; **(C)** heading date and **(D)** plant height.

Five inbred rice varieties representing different breeding objectives from the 1970s to the present were chosen. Ten F1 hybrids were obtained from five varieties using a diallele crossing design (Additional file 1: Figure S1 and Additional file 2: Figure S2). Three important agronomic traits related to grain yield (that is, plant height, heading date and grains per panicle) were evaluated based on the phenotypes of F1 hybrids from the diallele crossing populations (Figure 1A, B and C). The results revealed that the GCA scores for the two rice varieties 93–11 and TQ were positive, whereas negative GCA scores were observed for the varieties GL, ZS and AJ (Figure 1D). Further analysis revealed that the phenotypes of the three agronomic traits in the F1 hybrids were always similar to or higher than 93–11 and TQ, when either was used as one parent in the cross (Figure 1A-C and Additional file 1: Figure S1). In contrast, the phenotypes of F1 hybrids derived from GL (Figure 1), AJ and ZS (Additional file 1: Figure S1 and Additional file 2: Figure S2) were significantly different compared to 93–11 and TQ. The parental phenotypes were significantly correlated with the F1 hybrids crossed to 93–11 and TQ (*r >* 0.97, *P <* 3.80E-07) but less significantly with the F1 hybrids resulting from crosses to GL, ZS and AJ (*r =* 0.81, *P <* 1.33E-03) (Table 1). These results indicated that the GCAs for the three agronomic traits relevant to grain yield in parents 93–11 and TQ were significantly higher than those of the varieties GL, AJ and ZS.

**Table 1:** The correlation of phenotypes between each parent and their F1s

**Parents**	**r**	***P *** **value**
ZS	0.81	1.33E-03
AJ	0.81	1.27E-03
GL	0.81	1.26E-03
93-11	0.97	3.80E-07
TQ	0.99	2.06E-10

### The phenotype bias toward the positive GCA effect parent is attributable to transcriptome bias

**Figure 2:**
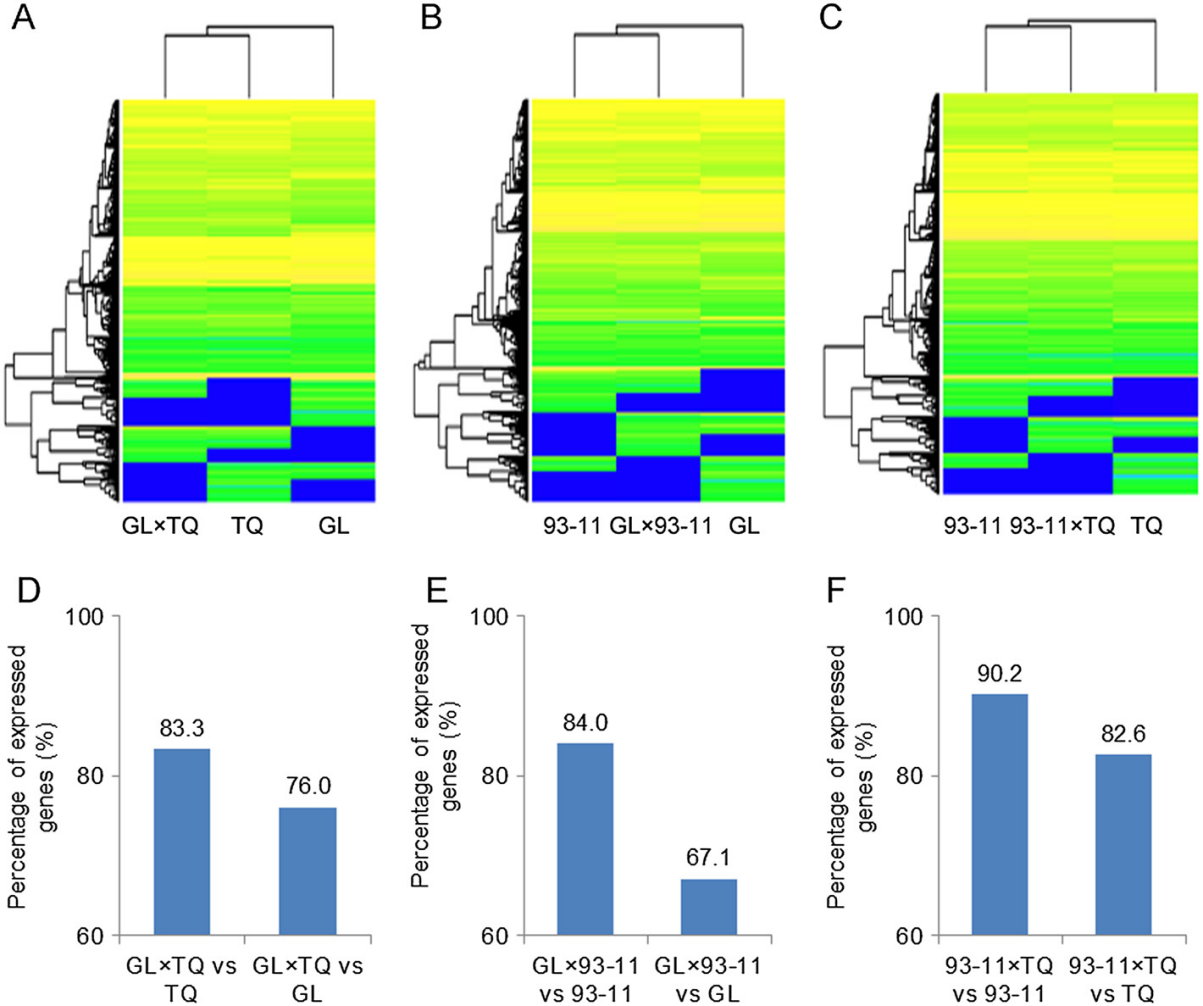
**Cluster analysis of global transcriptome of the three F1 hybrids and their parents.** Clusters of transcriptomes between GL, GL × TQ and TQ **(A)**; GL, GL × 93-11 and 93–11 **(B)** and 93–11, 93-11 × TQ and TQ **(C)**. The percentage of DEGs between F1 hybrids and the parents, GL × TQ **(D)**, GL × 93-11 **(E)** and 93-11 × TQ **(F)**.

As shown in Figure 1 and Additional file 3: Figure S3, GL had an earlier heading date (68.4 days on average) and had a shorter plant height (88.9 cm on average), whereas TQ and 93–11 had later heading dates (90.1 and 93.7 days on average) and had a taller plant height (113.3 and 117.2 cm on average, respectively). The heading dates (90.5 and 109.5 days on average, respectively) and plant heights (113.9 and 117.0 cm on average, respectively) in the F1 hybrids derived from TQ and 93–11 were obviously biased toward TQ or 93–11. To understand the relationship between the F1 phenotype and the positive GCA effect parent, we conducted a transcriptome profile analysis of the three parents, GL, 93–11 and TQ, and their three F1 hybrids, GL × 93-11, GL × TQ and 93-11 × TQ, using RNA sequencing technology (Additional file 3: Figure S3) [11]. A cluster analysis indicated that the transcriptomes of GL × TQ and GL × 93-11 are similar to those of the positive GCA effect parents, TQ and 93–11, respectively, and are significantly different from those of the negative GCA effect parent GL (Figure 2A and 2B). The transcriptome profile of 93-11 × TQ was more similar to 93–11 that had the higher positive GCA effect than to TQ (Figure 2C). The transcriptome similarity of the F1 hybrids to either the 93–11 or TQ parent was consistent with the phenotypes of the three traits in 93–11 and TQ. To explore the transcriptome profiles of the parents in the F1 hybrids, we analyzed the gene expression level in F1 hybrids. The results showed that 76.0% of the gene expression profiles in GL × TQ were similar to GL and 83.7% were similar to TQ (Figure 2D). Analogous results were found for the GL × 93-11 and 93-11 × TQ (i.e., 84% of the gene expression profiles in GL × 93-11 were similar to those in 93–11 and 67.1% of genes bias to GL) (Figure 2E). Of 90.2% expressed genes in 93-11 × TQ were similar to those in 93–11 and 82.6% of the genes bias to TQ (Figure 2F). Therefore, the phenotypes of the F1s derived from the positive GCA effect parents are correlated with a transcriptome bias in the F1s.

### Differentially expressed genes in the F1 hybrids are those preferentially expressed in the positive GCA effect parent

**Figure 3:**
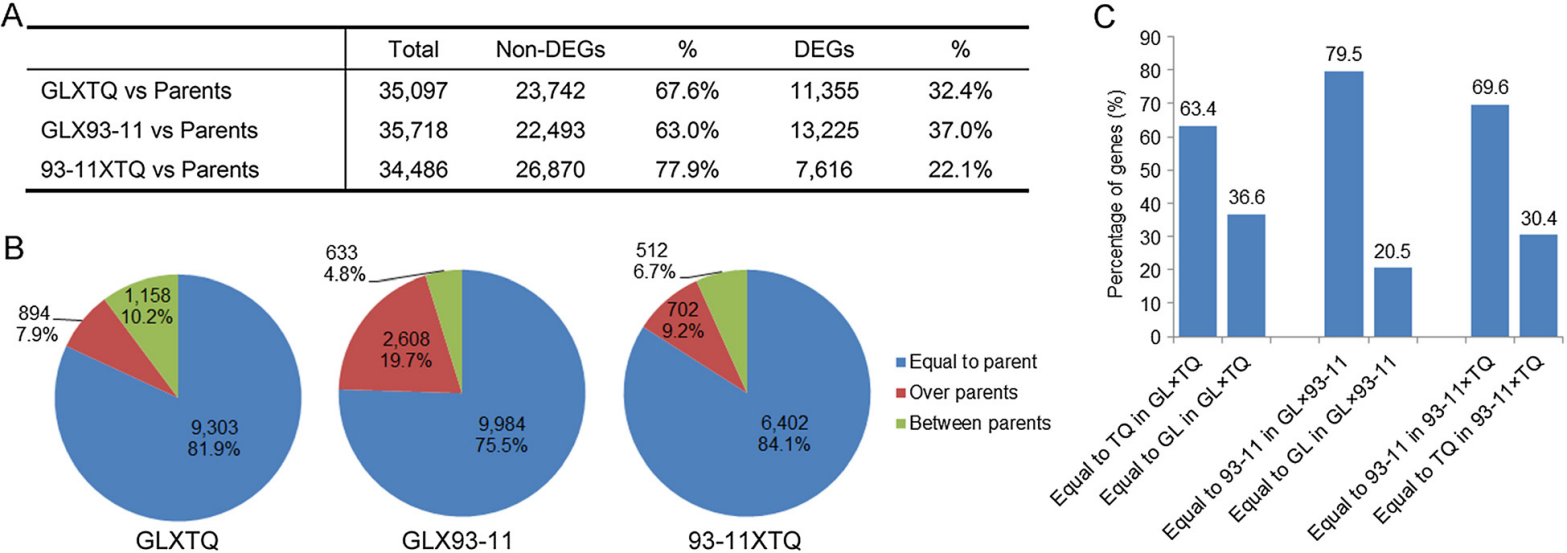
**Global differentially expressed genes between the F1 hybrids and their parents. (A)** The proportions of DEGs and non-DEGS were detected in three F1 hybrids; **(B)** categorization of different types of DEGs between F1 hybrids and parents. Equal to parent means the expression level difference in the F1 hybrids exists either one parent; over parents means the expression level in F1 the hybrids is out of the ranges of both parents; between parents means the difference of the expression level in F1 hybrids exists both parents, but not out of the parent range; and **(C)** Parent bias of the DEGs that were expressed at a level equal to one parent.

Differentially expressed genes (DEGs) have been recognized to play important biological functions in heterosis [16-18]. To explore whether DEGs contribute to the positive GCA effect parent in the F1 hybrids, we analyzed the DEG profiles from three F1 hybrids. A total of 34,486-35,718 genes were expressed in the F1 hybrids and their parents (Figure 3A). We found that 22.1% to 37.0% genes were differentially expressed in the F1 hybrids comparing GL × TQ, GL × 93-11 and 93-11 × TQ with their parents, respectively (fold change >2.0, *P <* 0.05) Moreover, 75.5% to 84.1% of the DEGs between the F1 hybrids and the parents had similar expression levels to one parent (Figure 3B). Of these, 63.4%, 79.5% and 69.6% of the gene expression levels were similar to the positive GCA effect parents TQ and 93–11 in GL × TQ, GL × 93-11 and 93-11 × TQ, respectively (Figure 3C). The expression levels of the remaining genes were similar to the negative or lower GCA effect parents GL and TQ (Figure 3C). 7.9% to 19.7% of the DEGs expression level in the F1 hybrids were out of the parental ranges, whereas 4.8% to 10.2% of the DEGs showed mid-parent expression levels, but showing significant difference to both parents (Figure 3B). These results reveal that the expression levels of the majority of the DEGs were similar to those of the positive GCA parent.

### Expression patterns of the genes in the regulatory pathways controlling flowering time and plant height in rice

To further support the observed correlation between the positive GCA effect phenotype in the parent and transcriptome bias in the F1, we chose the genes in two well-known signaling pathways controlling flowering time (Additional file 4: Table S1) and plant height in rice. The expression of *EARLY HEADING DATE1* (*Ehd1*) and *EARLY HEADING DATE2* (*Ehd2*) and repression of *HEADING DATE1* (*Hd1*), *HEADING DATE3a* (*Hd3a*) and *RICE FLOWERING LOCUS T1* (*RFT1*) are activated in a cascade to initiate rice flowering [19-25]. Expression of the Rice *Pseudo-Response Regulators 1* (*OsPRR1*) and *Grain number, plant height and heading date7* (*Ghd7*) involves a delay in the flowering time and an increase in the plant height and grain number [26-28]. We found that the expression levels of *OsPRR1*, *Hd1* and *Ghd7* were very high in TQ, 93–11, GL × TQ, GL × 93-11 and 93-11 × TQ versus GL (Table 2). By contrast, the expression levels of *RFT1*, *Hd3a*, *Ehd1* and *Ehd2* were higher in GL (Table 2). These results showed that the expression patterns of the flowering regulation genes were consistent with transcriptome bias towards the positive GCA effect parents in the F1 hybrids.

**Table 2:** Expression level of flowering- and GA metabolism-related genes

**Gene name**		**Expression level (RPKM)**				
	**GL**	**TQ**	**93-11**	**GLXTQ**	**GLX93-11**	**93-11XTQ**
Flowering pathway						
*OsPRR1*	35.49	92.53	122.96	122.26	89.91	138.95
*RFT1*	2.28	0.16	0.43	1.45	0.48	0.20
*Hd3a*	1.33	0.00	0.00	0.21	0.00	0.00
*Hd1*	0.57	3.27	1.30	2.17	5.82	1.62
*GHd7*	0	0	0.30	0	0.64	0.18
*EHd2*	2.05	0.83	0.22	1.08	0.81	0.32
*EHd1*	2.63	0	0	0	0	0
GA metabolism pathway						
*OsCPS1*	0.23	0.46	0.71	0.92	0.50	0.67
*OsKAO*	0.07	0.61	0.66	0.95	1.17	0.61
*OsGA3ox2*	2.12	0.10	0.22	0.80	0.46	0.92
*OsGA20ox2*	12.92	3.15	4.84	9.36	5.78	5.51
*OsGA2ox6*	0.82	5.43	1.12	4.54	8.37	7.95

The expression patterns of the other set of genes involved in gibberellic acid (GA) metabolism and the signaling pathway that controls plant height in rice were also analyzed between F1s and parents [29-32] (Additional file 5: Table S2). In plants, bioactive GAs are synthesized from the precursor geranylgeranyl diphosphate by ent-copalyl diphosphate synthase (CPS) and ent-kaurene synthase (KS), followed by ent-kaurene oxidase (KO) and ent-kaurenoic acid oxidase (KAO). At the final stage, GA_20_-oxidase (GA20ox) and GA_3_-oxidase (GA3ox) catalyze the conversion of GA_53_/GA_12_ and GA_1_/GA_4_. The bioactive GAs and the precursors elongated the uppermost internode and were deactivated by GA_2_-oxidase (GA2ox) and (EUI) [30]. As shown in Table 2, three genes, *ent-copalyl diphosphate synthase* (*OsCPS1*), *ent-kaurenoic acid oxidase* (*OsKAO*) and *GA*_2_*-oxidase 6* (*OsGA2ox6*) had the higher expression levels in TQ, 93–11, GL × TQ, GL × 93-11 than in GL. In contrast, expression levels of the other three genes, *GA3ox2* and *GA20ox2* were less in TQ, 93–11, GL × TQ, GL × 93-11 and 93-11 × TQ than in GL. The expression profiles of those genes exactly matched the feedback and feed-forward regulation mechanism of GAs synthesis [31,32]. Our results are consistence with the results reported in wheat hybrids [33,34]. The results again demonstrated that the expression patterns of the genes corresponding to plant height exhibited a bias toward the positive GCA effect parents. Taken together, the data validated the transcriptome bias toward the positive GCA effect parents through individual metabolism pathways for plant height and heading date in rice and further demonstrated that the phenotype bias to the positive GCA effect parent in F1 hybrids is due to the transcriptome bias toward the positive GCA effect parents.

### Transcriptome bias toward the positive GCA effect parent regulated by H3K36me3 modifications

**Figure 4:**
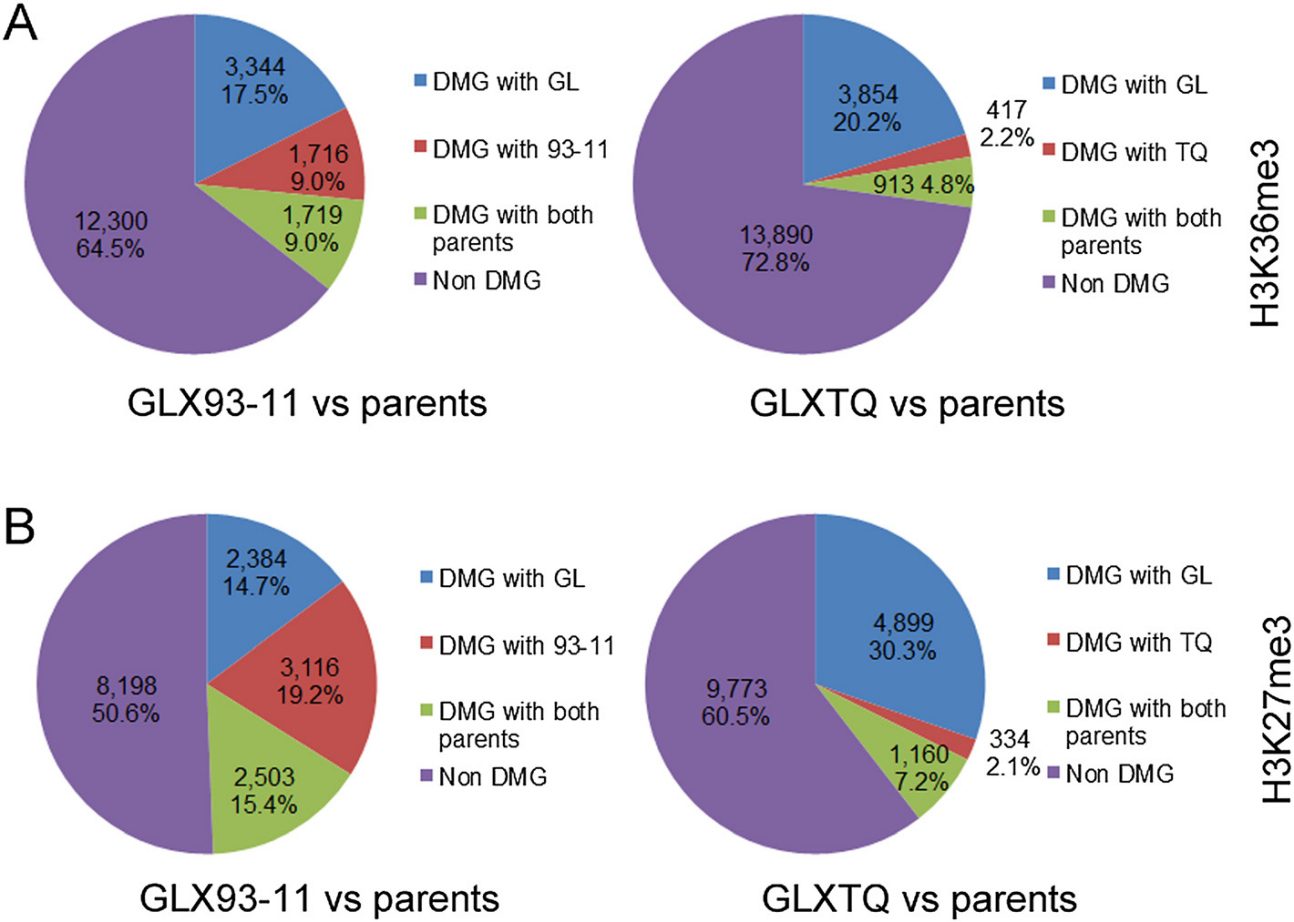
**The epigenetic modification level in F1 hybrids compared to each parent. (A)** Percentages of genes with H3K36me3 modification levels similar to those in each parent in F1 hybrids. **(B)** Percentages of genes with H3K27me3 modification levels similar to those of each parent in F1 hybrids.

Previous studies have described the histone modifications involved in the regulation of the transcriptome [35-38]. Previous studies have indicated that the trimethylation of histone H3 on lysine 27 (H3K27me3) in gene body represses gene expression [10,39,40] and trimethylated histone H3 on lysine 36 (H3K36me3) in the gene body activated gene expression [41-43]. To further explore the mechanism of transcriptome bias to the positive GCA effect parent in F1 hybrids, we analyzed the patterns of methylation at H3K36me3 and H3K27me3 in F1 hybrids. Genome-wide histone modifications of H3K36me3 and H3K27me3 in GL × 93-11, GL × TQ and their parents, GL, 93–11 and TQ, were analyzed with the aid of a DNA library prepared after chromatin immunoprecipitation (ChIP). The results showed 18.2%-18.3% genes were overlapped between H3K36me3 and H3K27me3 modifications. However, an obvious parental bias of H3K36me3 modification was detected. In GL × 93-11, 82.0% of the genes with H3K36me3 modifications were similar to 93–11 (FDR > 0.001), whereas 73.4% were similar to GL (FDR > 0.001) (Figure 4A). 93.0% of H3K36me3 modifications in GL × TQ were found to prefer TQ (FDR > 0.001), and 75.0% of H3K36me3 preferred GL (FDR > 0.001). For H3K27me3 modifications, 69.8% of the genes in GL × 93-11 with modifications were similar to GL (FDR > 0.001), and 65.2% were biased to 93–11 (FDR > 0.001) (Figure 4B). In GL × TQ, 90.7% genes with the H3K27me3 modifications preferred TQ (FDR > 0.001), whereas only 62.5% of H3K27me3 modifications were biased to GL (FDR > 0.001) (Figure 4B). There was no significant correlation of H3K27me3 between positive GCA effect and negative GCA effect parents in two F1 hybrids. Taken together, the results indicated that H3K36me3 modifications exhibited a bias toward the positive GCA effect parent in the F1 hybrids, which suggested that epigenetic regulation could involve regulation of the phenotypes and transcriptome bias toward positive GCA effect parents.

**Figure 5:**
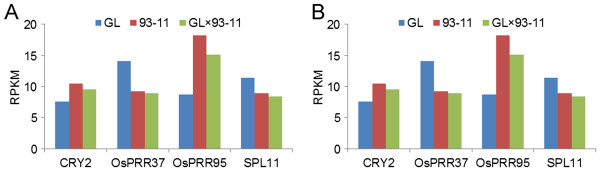
**H3K36me3 modification level of some flowering pathway genes between F1 hybrids and parents. (A)** H3K36me3 modification level between GL × 93-11 and the parents GL, 93–11. **(B)** H3K36me3 modification level between GL × TQ and the parents GL, TQ.

Furthermore, we analyzed the modification patterns of *Cryptochrome 2* (*CRY2*), *Pseudo-Response Regulator 37* and *95* (*PRR37* and *PRR95*) in F1 hybrids (Figure 5). These genes are involved in the regulation of rice flowering time and adaptability. *CRY2* is a photolyase-like blue-light receptor that mediates light responses in plants via interaction with the CIB1 (cryptochrome-interacting basic-helix-loop-helix) protein to promote *CRY2*-dependent floral initiation [44]. The *PRR37* gene is involved in the down-regulation of *Hd3a* gene expression to suppress flowering under long-day conditions. The varieties harboring nonfunctional alleles of *PRR37* flower extremely early under natural, long-day conditions [45]. We found that the H3K36me3 modification levels of three genes in GL × 93-11 or GL × TQ biased toward the positive GCA effect parents, TQ or 93–11 (Figure 5). The results are consistent with the heading date phenotype bias toward high GCA effect parents in the F1. Again, our data demonstrated that the phenotype and transcriptome biased toward the positive GCA effect parents were epigenetically regulated by both global modification and specific signaling pathways in rice.

## Discussion

The GCA concept has been studied for more than 70 years [1]. It has being successfully applied in crop and livestock breeding for the evaluation of parent performance [3-5]. However, in spite of this, the genetic analysis of the GCA is very limited [6,7], and the molecular mechanism of the GCA has not been documented. In the present study, we found that certain phenotypes in rice F1 hybrids were always biased toward the positive GCA effect parents in the traits tested (r > 0.97, *P <* 3.80E-07). Further studies showed that the transcriptome profiles in F1 hybrids were the same as phenotypes that are biased to the parents with positive GCA effect. The expression patterns of individual genes corresponding to plant height and flowering time demonstrated that the phenotype bias toward the positive GCA effect parent is attributed to the transcriptome profile and the specific epigenetic modification. Our data revealed that the phenotype bias in F1 hybrids could be attributed to transcriptome and H3K36me3 modification bias. Our findings provide molecular clues as to why and how the positive GCA effect parent has been widely used for crop and livestock improvement in breeding programs for half a century.

Although GCA is widely recognized and applied to crop and livestock improvement by breeders, it is based largely on breeding experiences, and therefore, it highly is unpredictable. Our findings provide molecular evidence of GCA through transcriptome and epi-genome analysis. This study first revealed that the transcriptome and epigenome in rice F1 hybrids are similar to the parent with the positive GCA performance, but significantly different from the negative GCA effect parents. We also found that the positive GCA effect parents showed better performance with regard to agronomic traits in F1 hybrids compared to the negative GCA effect parents. In the previous studies, a large number of genetic loci with dominance effect were detected in F1 hybrids [46-48]. eQTL analysis revealed that the gene expression regulation was an complicated regulation networks [49-51]. So, the phenomena of the phenotypes and the transcriptomes biased to high GCA parents in F1 hybrids might be consequence of the accumulation of the loci with dominance effect in the elite parents through artificial selection. Our results suggested that the transcriptomes profile in F1 hybrids could be optimized during elite parent selection, which give rise to elite agronomic traits, such as increase of biomass and grain yield. During the parent improvement process, these alleles corresponding to the agronomic traits satisfying different breeding objectives had been selected or kept. For instance, a representative elite variety of GL that met the breeding objectives of more panicle and less grain number per panicle had been developed in the 1970s in China. Later, an ideal plant type that has less panicle numbers and more spikelet per panicle was raised, and the breeding objectives of higher grain yields required high biomass that was due to later heading date and higher plant height. To meet the requirements of the breeding objective, the alleles corresponding to late heading date and higher plant height were accumulated and optimized through artificial selection pressure. Therefore, the modern elite parent 93–11 has a longer flowering time and taller plant height that produces higher biomass resulting in higher grain yield. Consequently, the alleles corresponding to later flowering time and taller plant height were selected and maintained in the genome of the elite parent. Our results again demonstrated the significance of selecting elite parents with excellent GCA performance in crossbreeding program.

Several studies have indicated that the mechanism of the allelic-specific expression could be regulated by epigenetic modification, including DNA methylation and various histone modifications [52,53]. Previous studies have detected maternal alleles for some imprinted genes were hypomethylated in the endosperm, which contributed to the parent-of-origin expression pattern in reciprocal hybrids in *maize*[52,53]. However, very few parent-of-origin effects were detected in plant vegetative tissues [54]. Our global gene expression analysis has not found any parent-of-origin genes, but almost of genes expression in rice leaves showed as genotype-dependent fashion [11]. In present work, the specific genes involved in plant height and circadian rhythms are matched to phenotype bias as well, suggesting that epigenetic regulation is highly selective and complicated. Limited data obtained in the present work may have led to finding the H3K36me3 modification only on the partial genes that were associated with the GCA effect, and these modifications did not match the expression level of the genes analyzed. The results of H3K27me3 modification profile biased to TQ in GL × TQ, but not bias in another F1 hybrids GL × 93-11 suggested that epigenetic modifications could be dynamic changes depending on genetic backgrounds, developmental stage, environment etc. Therefore, more research into epigenetic modifications, such as other histone modifications, acetylation and DNA methylation, are needed to fully understand the genome-wide regulation network.

Extensive studies have revealed that a large number of genes exhibited differential expression level between F1 hybrids and their parents [8,10,12,13,55,56]. However, the regulation of gene expression profiles in the heterozygous state remains unknown. Our data revealed that the positive GCA effect parent in the F1 hybrids could determine the phenotypes of the important agronomic traits, such as plant height and heading date. Plant height and heading date are very important agronomic traits that affect rice grain yield [27,57]. Regulation of the genes in the signal transduction pathway and syntheses of metabolic products have been reported to occur through SNPs in these gene sequences that could be optimized to the gene expression profiles in elite varieties [21]. Our results indicated that the phenotypes of parental bias in F1 hybrids could be the consequence of transcriptome profile or some specific gene expression level.

One of the most important characteristics of the elite parent with high GCA effect is its wide adaptability. The wide adaptability of the elite parent could be due to both photoperiod and temperature neutral in order to adapt different ecological environments. Therefore, rice plant could maintain a constant growth period so that it can develop higher biomass and grain yield in different ecological environments. Previous study has reported the *OsPRR37* had involved in the regulation network of the adaptability to grow in different latitudes [45,57]. In present work, we found the H3K36me3 modification patterns of the *OsPRR* gene family, *OsPRR37* and *OsPRR95,* showed similar patterns in F1 hybrids, high GCA parents 93–11 and TQ. The results indicated that the specific modification patterns of H3K36me3 in high GCA parents might contribute to the adaptability for elite parents and their offsprings. It is reasonable to assume that the *PRR* gene family could play an important role in controlling the adaptability of an elite parent that is responsible for GCA effects in rice.

## Conclusions

The results presented here revealed that the transcriptome profiles and DEGs in the F1s were highly related to phenotype bias to the positive GCA-effect parent. The transcriptome bias was also demonstrated by analyzing the genes that controlling the specific phenotypes of plant height and flowering and H3K36me3 modification on global modification or specific signaling pathways. Our results indicated the molecular basis of GCA is both on transcriptome and epigenetic level.

## Methods

### Plant material and phenotype analysis

Five rice elite varieties, GL, TQ, 93–11, AJ and ZS, were chosen for this study. Their pedigree as showed in Additional file 6. GCA evaluation was conducted by a diallele crossing design. Briefly, 10 parallel crosses derived from five parents and the phenotypes were collected from a field in Wuhan in 2008 and 2009 and from the Hainan Island province in 2009. The plots were established in triplicate with 30 plants per plot in every season. Three agronomic traits, plant height (PH), heading date (HD) and grain number per panicle (GNP), were studied. The general combining ability (GCA) for each parent was calculated according to Griffing [58].

### Nuclear RNA extraction

The second fully expanded leaves were harvested at the secondary branch differentiation stage, immediately frozen in liquid nitrogen and stored at -80°C. The leaves from the triplicate plots were pooled for RNA extraction. Nuclei were isolated from ~10 g of frozen leaves using the Plant Nuclei Isolation/Extraction Kit (Sigma, St. Louis, MO, USA). Total hnRNA was extracted from nuclei using Trizol (Invitrogen, Carlsbad, CA, USA) according to the manufacturer’s instructions, and then treated with RNase-free DNase I (New England Biolabs, Ipswich, MA, USA) to remove any contaminating genomic DNA.

### RNA-Seq Library construction

The Illumina mRNA-Seq Sample Prep Kit (Illumina, San Diego, CA USA) was used to prepare the sequencing library with 3 μg of nuclear RNA. Fragmentation buffer in the kit was added directly to hnRNA to produce short fragments of 200–700 bp, which served as the templates for first-strand cDNA synthesis using random hexamers. Second-strand cDNA was synthesized followed the protocol described in the kit and was purified using a QIAquick PCR Extraction Kit (Qiagen, Valencia,CA USA) and eluted in elution buffer (EB). The short fragments were then ligated to sequencing adapters. Suitable fragments of approximately 200 bp were selected as templates for amplification in a MyCycler PCR instrument (Bio-Rad, Hercules, CA USA) with the following program: denaturation at 98°C for 30 s followed by 15 cycles of 98°C for 10 s, 65°C for 30 s, and 72°C for 30 s plus a terminal hold at 72°C for 5 min. The samples were then purified using the QIAquick PCR Purification Kit according to the manufacturer’s protocol and eluted in 30 μL of EB. One μL aliquot of the construct was loaded onto an Agilent Technologies 2100 Bioanalyzer using the Agilent DNA 1000 Chip Kit (Agilent, Santa Clara, CA USA). After verifying the size and purity of the DNA fragments, the library was sequenced using an Illumina GA II x platform by BGI (Shenzhen, China).

### ChIP-Seq library generation

Chromatin immunoprecipitation (ChIP) was performed with antibodies against trimethylated histone H3 on lysine 27 (H3K27me3, Abcam Cat. #ab6002, Cambridge, MA, USA) and trimethylated histone H3 on lysine 36 (H3K36me3) (Abcam, Cat. #ab9050, Cambridge, MA, USA) as described by Saleh et al. [59]. The DNA was extracted by adding equal volumes of phenol/chloroform/isoamyl alcohol to each tube and vortexing briefly. The DNA was precipitated with 2.5 volumes of 100% EtOH, 1/10 volume of 3 M sodium acetate (pH 5.2) and 2 μl of glycogen (20 mg ml^-1^) at -80°C. Next, the ChIP DNA was used to generate Illumina sequencing libraries following the manufacturer’s protocol of Paired-End DNA Sample Prep Kit (Catalog #: PE-102-1001, Illumina, San Diego, CA, USA): appropriate fragments approximately 200 base pairs (bp) in length were selected as templates for amplification involving denaturation at 98°C for 30 s; 15 cycles of 98°C for 10 s, 65°C for 30 s and 72°C for 30 s; and a final incubation at 72°C for 5 min. The samples were then purified using the QIAquick PCR Purification Kit according to the manufacturer’s protocol and were eluted in 30 μl of elution buffer. One microliter of the library was loaded on an Agilent Technologies 2100 Bioanalyzer using the Agilent DNA 1000 chip kit (Agilent, part #5067–1504). After its size and purity were verified, the library was sequenced using the Illumina GAIIx platform.

### Analysis of ChIP-Seq reads with the Reads Per Kb per Million reads (RPKM) method

Based on the gene body-specific distributions of both H3K27me3 and H3K36me3 [40,60][10], we normalized the ChIP-Seq read counts by calculating the number of RPKMs in the gene body region.

### Statistical analysis

A threshold of more than a 2-fold difference in gene expression levels and a False Discovery Rate (FDR) of less than 0.05 were used to identify the DEGs. An FDR less than 0.001 were used to identify the genes differentially modifying histones. The evaluation of the *P* value and FDR of DEGs was performed as described by Audic et al. [61] and Benjamini et al. [62], respectively. The *t*-test and correlation analysis were conducted using Microsoft Office Excel 2010.

### Quantitation real time PCR

The confirmation of gene expression level by qRT-PCR were described by Song et al. [11], and the methods were described by Wang et al. [63].

## Abbreviations

GCA: General combining ability; SCA: Specific combining ability; DEG: Differentially expressed gene; FDR: False discovery rate; RPKM: Reads Per Kb per Million reads.

## Competing interests

The authors declare that they have no competing interests.

## Authors’ contributions

GY, S performed the most of the experiments; ZB, G performed the epigenetics experiments; DM, J performed all field experiments; ZW, L, XF, Q, W, W, helped GY, S performed crossing and analysis of phenotypes; DC, Y and YG, Z supervised this study; DC, Y and GY, S designed the experiments and wrote the manuscript. All the authors discussed the results and contributed to the manuscript. All authors read and approved the final manuscript.

## Supplementary Material

Additional file 1: Figure S1 Phenotypes related to grain yields from 10 diallele crosses. Click here for file

Additional file 2: Figure S2 Plant morphologies of 10 diallele crosses. Click here for file

Additional file 3: Figure S3 Plant phenotypes of three parents and their F1 hybrids used for transcriptome sequencing. Click here for file

Additional file 4: Table S1 The expression levels of the known genes involved in the flowering pathway in rice. Click here for file

Additional file 5: Table S2 The expression level of the known genes involved in GA metabolism and signaling pathways in rice. Click here for file

Additional file 6: Figure S4 The pedigree of five parents used in this study. The pedigree of five parents was constructed by 153 polymorphism SSR markers using the Ntsys2.1 software. Click here for file
