# Supplementary figures and images for: The phenotypic predisposition of the parent in F1 hybrid is correlated with transcriptome preference of the positive general combining ability parent
Source: BMC Genomics. 2014 Apr 22;15:297. doi: 10.1186/1471-2164-15-297 (PMC4023606; doi:10.1186/1471-2164-15-297)

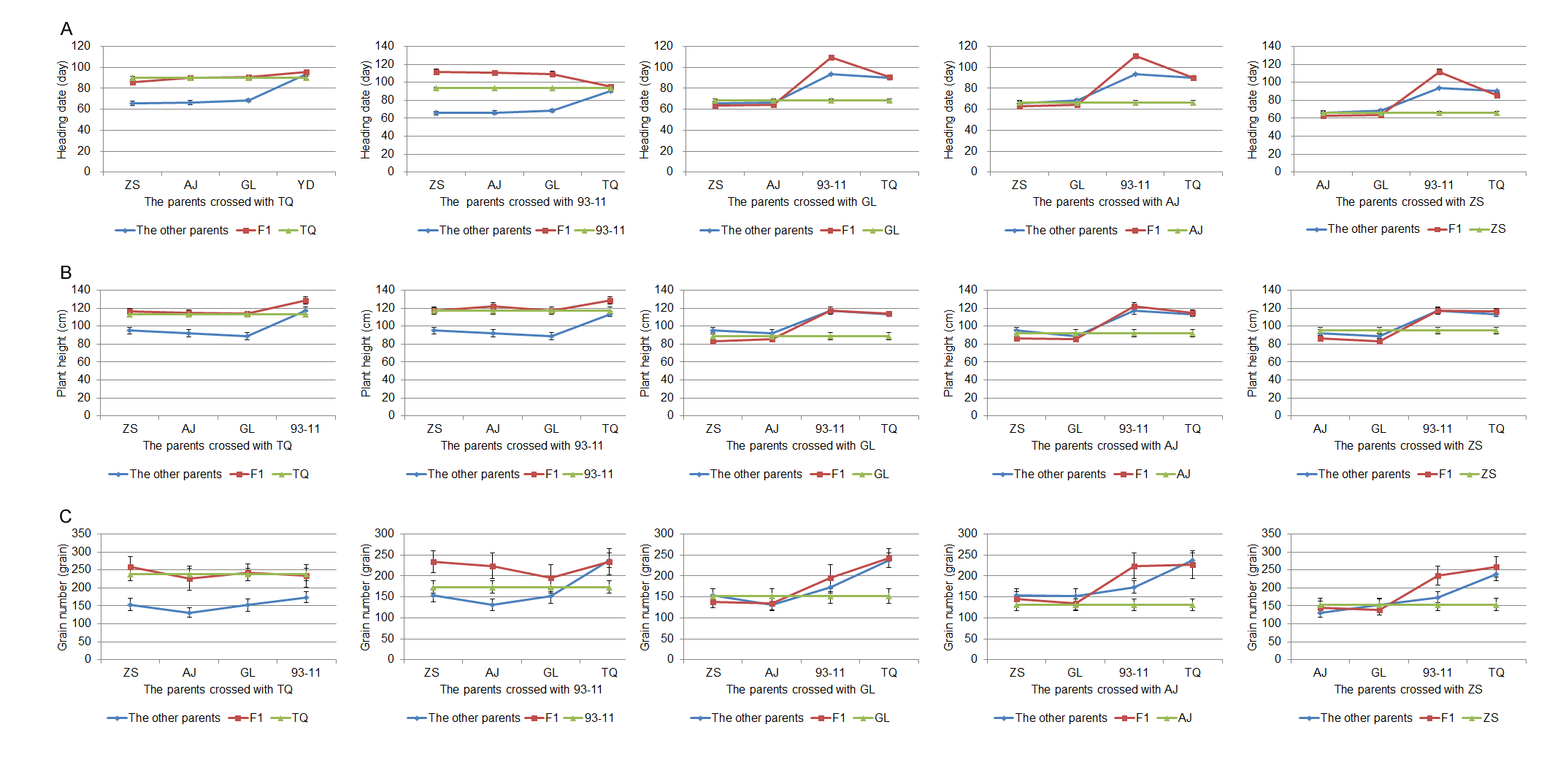

Supplement: Additional file 1: Figure S1 — Phenotypes related to grain yields from 10 diallele crosses. [file 1471-2164-15-297-S1.tiff]

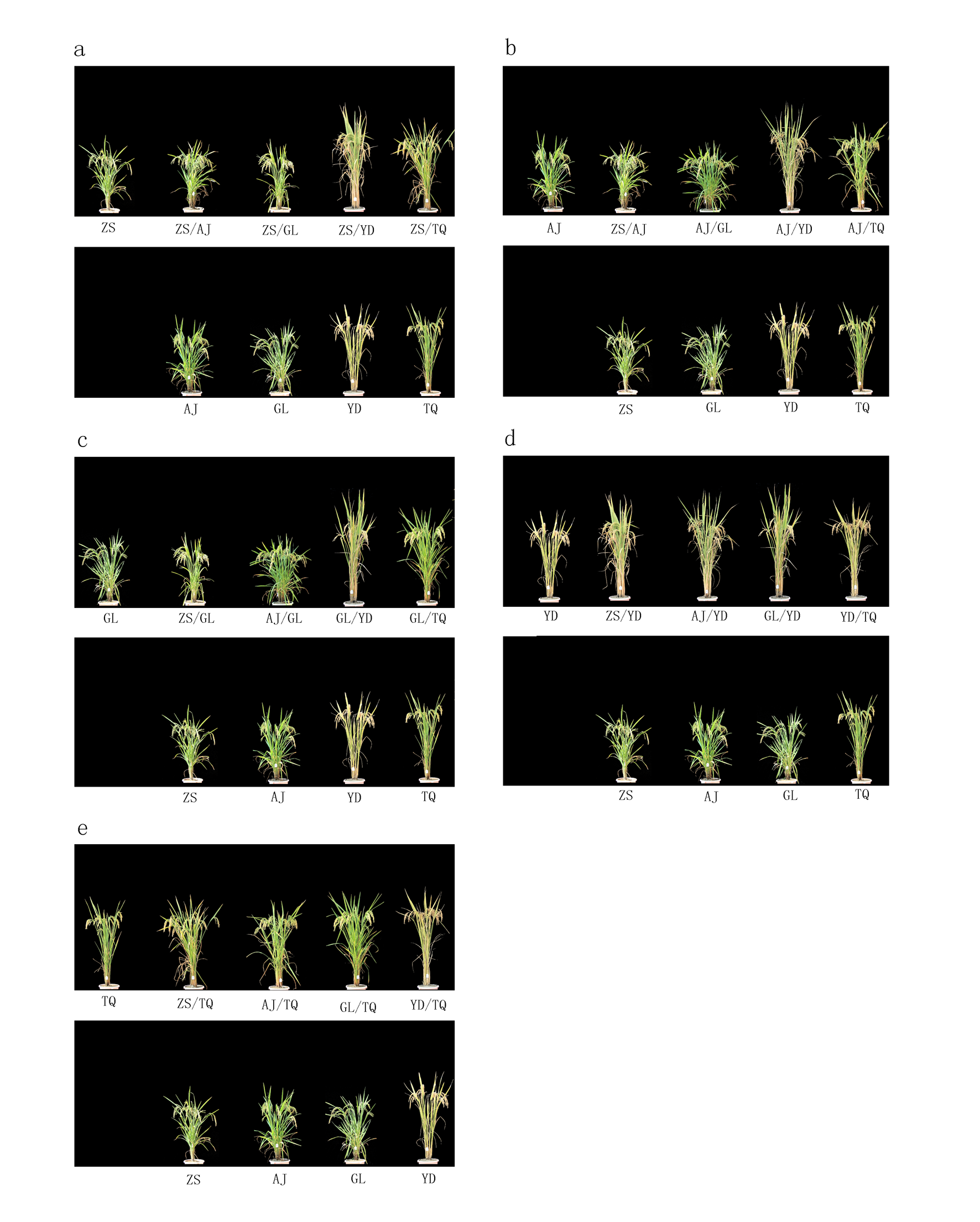

Supplement: Additional file 2: Figure S2 — Plant morphologies of 10 diallele crosses. [file 1471-2164-15-297-S2.tiff]

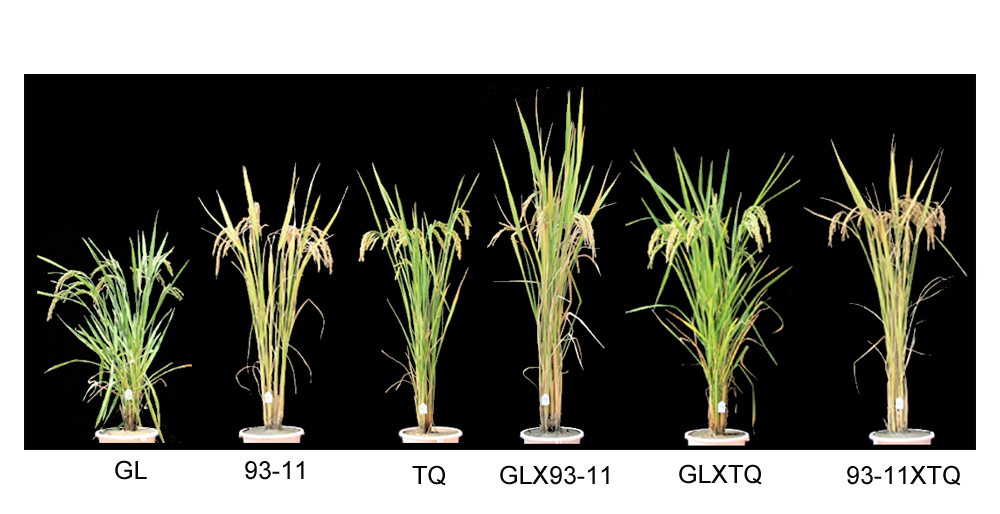

Supplement: Additional file 3: Figure S3 — Plant phenotypes of three parents and their F1 hybrids used for transcriptome sequencing. [file 1471-2164-15-297-S3.tiff]

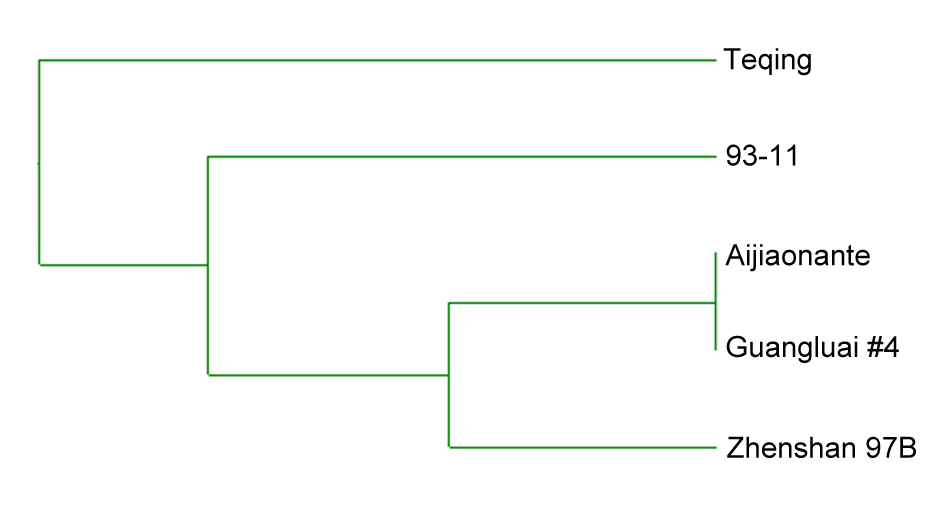

Supplement: Additional file 6: Figure S4 — The pedigree of five parents used in this study. The pedigree of five parents was constructed by 153 polymorphism SSR markers using the Ntsys2.1 software. [file 1471-2164-15-297-S6.tiff]
